# Chest CT findings in severe acute respiratory distress syndrome requiring V-V ECMO: J-CARVE registry

**DOI:** 10.1186/s40560-023-00715-x

**Published:** 2024-01-26

**Authors:** Mitsuaki Nishikimi, Shinichiro Ohshimo, Wataru Fukumoto, Jun Hamaguchi, Kazuki Matsumura, Kenji Fujizuka, Yoshihiro Hagiwara, Ryuichi Nakayama, Naofumi Bunya, Junichi Maruyama, Toshikazu Abe, Tatsuhiko Anzai, Yoshitaka Ogata, Hiromichi Naito, Yu Amemiya, Tokuji Ikeda, Masayuki Yagi, Yutaro Furukawa, Hayato Taniguchi, Tsukasa Yagi, Ken Katsuta, Daisuke Konno, Ginga Suzuki, Yuki Kawasaki, Noriyuki Hattori, Tomoyuki Nakamura, Natsuki Kondo, Hitoshi Kikuchi, Shinichi Kai, Saaya Ichiyama, Kazuo Awai, Kunihiko Takahashi, Nobuaki Shime, J-CARVE registry group, Junki Ishii, Takayuki Ogura, Mitsunobu Nakamura, Keiki Shimizu, Tatsutoshi Shimatani, Mamoru Masuda

**Affiliations:** 1 Department of Emergency and Critical Care Medicine, Graduate School of Biomedical and Health Sciences, Hiroshima University, 1-2-3 Kasumi, Minami-ku, Hiroshima, 7348551 Japan; 2 Department of Diagnostic Radiology, Graduate School of Biomedical and Health Sciences, Hiroshima University, Hiroshima, Japan; 3 Department of Critical Care and Emergency Medicine, Tokyo Metropolitan Tama Medical Center, Tokyo, Japan; 4 Advanced Medical Emergency Department and Critical Care Center, Japan Red Cross Maebashi Hospital, Maebashi, Japan; 5 Department of Emergency Medicine and Critical Care Medicine, SAISEIKAI Utsunomiya Hospital, Utsunomiya, Japan; 6 Department of Emergency Medicine, Sapporo Medical University, Sapporo, Japan; 7 Department of Emergency Medicine and Critical Care, Fukuoka University Hospital, Fukuoka, Japan; 8 Department of Emergency and Critical Care Medicine, Tsukuba Memorial Hospital, Tsukuba, Japan; 9 Department of Biostatistics, M&D Data Science Center, Tokyo Medical and Dental University, Tokyo, Japan; 10 Department of Critical Care Medicine, Yao Tokushukai General Hospital, Osaka, Japan; 11 Department of Emergency, Critical Care and Disaster Medicine, Okayama University Graduate School of Medicine, Dentistry and Pharmaceutical Sciences, Okayama, Japan; 12 Department of Emergency and Critical Care Medicine, Osaka Medical and Pharmaceutical University, Osaka, Japan; 13 Department of Emergency Medicine and Critical Care Medicine, Yamanashi Prefectural Central Hospital, Kofu, Japan; 14 Emergency Medical and Acute Care Surgery, Matsudo City General Hospital, Matsudo, Japan; 15 Advanced Critical Care Center, Saga University Hospital, Saga, Japan; 16 Advanced Critical Care and Emergency Center, Yokohama City University Medical Center, Yokohama, Japan; 17 Department of Emergency and Critical Care Medicine, Nihon University Hospital, Tokyo, Japan; 18 Department of Emergency and Critical Care, Tohoku University Hospital, Sendai, Japan; 19 Department of Anesthesiology and Perioperative Medicine, Tohoku University School of Medicine, Sendai, Japan; 20 Emergency and Critical Care Center, Toho University Omori Medical Center, Tokyo, Japan; 21 Department of Emergency and Disaster Medicine, Gifu University Graduate School of Medicine, Gifu, Japan; 22 Department of Emergency and Critical Care Medicine, Chiba University Graduate School of Medicine, Chiba, Japan; 23 Department of Anesthesiology and Critical Care Medicine, Fujita Health University School of Medicine, Toyoake, Japan; 24 Department of Intensive Care, Chiba Emergency Medical Center, Chiba, Japan; 25 Department of Emergency Medicine, Koga Community Hospital, Yaizu, Japan; 26 Department of Emergency Medicine, Sagamihara Kyodo Hospital, Sagamihara, Japan; 27 Department of Anesthesia, Kyoto University Hospital, Kyoto, Japan; 28 Department of Emergency and Disaster Medicine, Hirosaki University, Hirosaki, Japan

**Keywords:** Computed tomography, In-hospital mortality, Static lung compliance, Traction bronchiectasis, Subcutaneous emphysema

## Abstract

**Background:**

Chest computed tomography findings are helpful for understanding the pathophysiology of severe acute respiratory distress syndrome (ARDS). However, there is no large, multicenter, chest computed tomography registry for patients requiring veno-venous extracorporeal membrane oxygenation (V-V ECMO). The aim of this study was to describe chest computed tomography findings at V-V ECMO initiation and to evaluate the association between the findings and outcomes in severe ARDS.

**Methods:**

This multicenter, retrospective cohort study enrolled patients with severe ARDS on V-V ECMO, who were admitted to the intensive care units of 24 hospitals in Japan between January 1, 2012, and December 31, 2022.

**Results:**

The primary outcome was 90-day in-hospital mortality. The secondary outcomes were the successful liberation from V-V ECMO and the values of static lung compliance. Among the 697 registry patients, of the 582 patients who underwent chest computed tomography at V-V ECMO initiation, 394 survived and 188 died. Multivariate Cox regression showed that traction bronchiectasis and subcutaneous emphysema increased the risk of 90-day in-hospital mortality (hazard ratio [95% confidence interval] 1.77 [1.19–2.63], *p* = 0.005 and 1.97 [1.02–3.79], *p* = 0.044, respectively). The presence of traction bronchiectasis was also associated with decreased successful liberation from V-V ECMO (odds ratio: 0.27 [0.14–0.52], *p* < 0.001). Lower static lung compliance was associated with some chest computed tomography findings related to changes outside of pulmonary opacity, but not with the findings related to pulmonary opacity.

**Conclusions:**

Traction bronchiectasis and subcutaneous emphysema increased the risk of 90-day in-hospital mortality in patients with severe ARDS who required V-V ECMO.

**Supplementary Information:**

The online version contains supplementary material available at 10.1186/s40560-023-00715-x.

## Background

Acute respiratory distress syndrome (ARDS) is a heterogeneous syndrome [1, 2], and the characteristics of “bilateral opacities”, according to the Berlin criteria for the definition of ARDS [3] differ from patient to patient [4–6]. ARDS can be classified under three severity categories [7]. The mortality rate of the most severe category—severe ARDS (PaO_2_/F_i_O_2_ ratio [P/F ratio] ≤ 100)—exceeds 50%, even if respiratory support with veno-venous extracorporeal membrane oxygenation (V-V ECMO) is attempted [8]. Considering this extremely high risk of mortality, studies focusing on severe ARDS requiring V-V ECMO support should be encouraged, although many previous studies, except for the Extracorporeal Life Support Organization registry analyses [8, 9], have been limited by small sample sizes [10, 11]. Analyses of data from a large multicenter database with novel findings may lead to the development of a new treatment strategy, including a more appropriate indication for V-V ECMO support, in this research field.

Therefore, we developed a retrospective database of patients with severe ARDS receiving V-V ECMO, named the Japan Chest CT for ARDS requiring V-V ECMO registry (J-CARVE registry), including data from 24 institutions across Japan. The J-CARVE registry is unique compared with other registries because it includes the chest computed tomography (CT) imaging data at V-V ECMO support initiation. Undoubtedly, chest CT findings are helpful in understanding the pathophysiology of ARDS [12, 13]; however, few studies have described the characteristics of chest CT findings in severe ARDS with V-V ECMO. This study aimed to describe the chest CT findings at the initiation of V-V ECMO support in patients with severe ARDS and evaluate the association between these findings and the risk of mortality.

## Methods

### Study design

Using data from the intensive care units (ICUs) at 24 institutions across Japan, we developed the J-CARVE registry—a retrospective database of patients with severe ARDS on V-V ECMO. Institutions that intended to participate in this registry had to submit a participation form available on the J-CARVE registry website (https://www.ace-registry.net) and needed to have treated at least 10 patients with severe ARDS who required V-V ECMO. The study was registered in the University Hospital Medical Information Network Clinical Trials Registry (UMIN-CTR) before starting data collection (UMIN000048709). The registry was approved by the Institutional Review Board of Hiroshima University Hospital (E-2768), which waived the requirement for obtaining informed patient consent to ensure participant anonymity, as stipulated in the Japanese government guidelines. The details of the J-CARVE registry data collection and quality control are described in Additional file 1: SMethods.

### Participants

The J-CARVE registry retrospectively enrolled adult (age ≥ 18 years) patients with severe ARDS for whom V-V ECMO support was initiated between January 2012 and December 2022. Severe ARDS was diagnosed based on the Berlin definition criteria (P/F ratio ≤ 100) [3]. In this study, patients were further excluded if they did not undergo chest CT examinations within a stipulated time window of 3 days from the start of V-V ECMO support or if the radiologists judged that the findings of the chest CT did not indicate ARDS.

### Interpretation of chest CT scans

The chest CT findings were interpreted by five Japanese board-certified radiologists (WF, KN, HM, SK, and SM), all of whom had > 5 years of experience in interpreting chest CT images of patients with ARDS, based on a previous study [14]. The representative images for each finding are shown in Additional file 1: Fig. S1. For each CT data, two reviewers were randomly selected to interpret the images in a blinded manner. Prior to the interpretation of any CT findings, the diagnosis of ARDS was confirmed radiologically, and patients were only excluded from further assessments if both radiologists determined that there were no findings indicative of ARDS.

The concordance rates between the two evaluators for each chest CT finding are summarized in Additional file 1: Table S1. Any disagreements were resolved by a third reviewer in a blinded manner. Two patients had a history of right upper lobectomy and were regarded as having no signs in the right upper lobe.

### Outcomes

The primary outcome was 90-day in-hospital mortality. The secondary outcomes were the successful liberation from V-V ECMO and values of static lung compliance.

### Statistical analyses

The Chi-square and Mann–Whitney *U* tests were used to compare categorical and continuous variables, respectively. For the survival analysis, the survival time (number of days from V-V ECMO support initiation until death or the last follow-up) was considered uncensored if the patient died in the hospital on day 90 or earlier. Survival times were censored at the date of hospital discharge or day 90, whichever occurred first. Adjusted Cox proportional hazards regression analyses were performed using several adjusted variables. Besides information regarding basic demographics and comorbidities (age, sex, body mass index, and medical histories of hypertension, diabetes, chronic kidney disease, obstructive lung disease, interstitial lung disease, and chronic heart failure), we analyzed the following variables: time from the start of mechanical ventilation (MV) to V-V ECMO initiation, Sequential Organ Failure Assessment score at the start of V-V ECMO support as an indicator of the patients’ clinical severity, application of prone positioning before V-V ECMO support, and use of neuromuscular blockers before V-V ECMO support. These variables were reported to be associated with the mortality of patients with ARDS on V-V ECMO [10, 15]. We performed subgroup analyses according to the duration from the start of MV and V-V ECMO support initiation (early vs. late induction) or the primary cause of ARDS (bacterial, viral, or other pneumonia). We set 7 days as the cut-off value because many previous studies used 7 days [16–19]; in addition, patients receiving MV for > 7 days before V-V ECMO showed a higher mortality rate than those receiving MV for < 7 days [16].

The multivariate logistic regression analysis was performed to evaluate the association between each chest CT finding and successful liberation from V-V ECMO using the same adjustment factors as those for the survival analysis. The association between each chest CT finding and static lung compliance was also evaluated using the Mann–Whitney *U* test. All reported *p*-values were two-sided, and *p* < 0.05 indicated statistical significance. All analyses were performed using the R Package (R Foundation for Statistical Computing, Vienna, Austria).

## Results

**Fig. 1:**
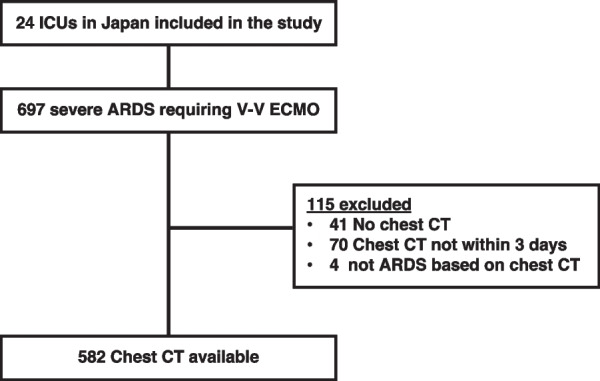
Flow diagram of patients. ARDS: acute respiratory distress syndrome; ICU: intensive care unit; V-V ECMO: veno-venous extracorporeal membrane oxygenation

The patient flow diagram of this study is shown in Fig. 1. Among 697 patients who were admitted in 24 ICUs in Japan, 115 were excluded for the following reasons: 41 patients had unavailable chest CT images, 70 underwent chest CT examinations but not close to the start of V-V ECMO support (within 3 days), and 4 were judged as not having ARDS based on the radiologists’ interpretation of their chest CT findings. The remaining 582 patients were analyzed. The distribution of registered patients by years is shown in Figure E2. The time difference between chest CT examinations and the start of V-V ECMO support among the analyzed patients is shown in Additional file 1: Fig. S3. In approximately 80% of all analyzed patients, the CT examination was performed within 24 h, and 90% underwent the chest CT within 48 h.

Additional file 1: Table S2 shows the basic information of all participating institutes. Among the 24 participating hospitals, 14 (58.3%) were academhospitalsic , whereas the remaining 10 (41.7%) were nonacademic hospitals. Eleven (45.8%) of all institutes routinely employed an ultra-lung-protective strategy of a limited tidal volume of ≤ 3 mL/kg during V-V ECMO support. The usual setting values of positive end-expiratory pressure during V-V ECMO support varied from 5 to 15 cmH_2_O among hospitals.

Baseline characteristics from before and after V-V ECMO are summarized in Tables 1 and 2, respectively. With regard to the primary cause of ARDS, 93.5% (544/582) were intrapulmonary and 6.5% (38/582) were extrapulmonary cases. The mean duration (days) between the start of MV and V-V ECMO instauration was 2.0 (1.0–4.0) days, the mean value of the P/F ratio was 89.1 (± 37.0), and the mean score point of the Murray Lung Injury Score was 3.25 (2.75–3.50) (Table 1). The average setting values of positive end-expiratory pressure and dynamic driving pressure during days 1–3 after V-V ECMO support were 10.0 (± 3.4) and 10.0 (± 5.2) cmH_2_O, respectively, whereas the corresponding values during days 4–7 were 10.3 (± 5.2) and 10.0 (± 5.0) cmH_2_O, respectively (Table 2). The median length of hospital stay was 35.0 (19.0–61.0) days. The proportion of patients who were successfully liberated from V-V ECMO was 82.2% (475/582). The median duration of V-V ECMO support was 11.0 (7.0–19.0) days. Regarding the 90-day in-hospital mortality, 175 (30.1%) patients died within 90 days after the initiation of V-V ECMO support, whereas 407 (69.9%) survived. ICU mortality among all analyzed patients was 29.0% (169/582).

**Table 1:** Baseline characteristics of all participants before V-V ECMO initiation

	All patients (*n* = 582)	Survived (*n* = 407)	Died (*n* = 175)	*p*-value
Age, years	60.0 (50.0–68.0)	58.0 (49.0–66.0)	65.0 (57.0–70.0)	< 0.001
Sex, male, *n* (%)	439 (75.4)	312 (76.7)	127 (72.6)	0.30
BMI, kg/m^2^^a^	25.7 (22.5–30.1)	26.0 (22.8–30.6)	25.0 (21.5–28.3)	0.012
Past medical history				
Hypertension, *n* (%)	227 (39.0)	158 (38.8)	69 (39.4)	0.89
Diabetes, *n* (%)	182 (31.3)	139 (34.2)	43 (24.6)	0.021
Chronic kidney disease, *n* (%)	48 (8.3)	32 (7.9)	16 (9.1)	0.61
Obstructive lung disease, *n* (%)	82 (14.1)	55 (13.5)	27 (15.4)	0.55
Interstitial lung disease, *n* (%)	23 (4.0)	13 (3.2)	10 (5.7)	0.16
Chronic heart failure, *n* (%)	33 (5.7)	20 (4.9)	13 (7.4)	0.24
Duration between MV and ECMO initiation, days	2.0 (1.0–4.0)	2.0 (1.0–3.0)	2.0 (1.0–7.0)	< 0.001
Primary etiology for ARDS, *n* (%)				0.18
Pulmonary	544 (93.5)	382 (93.4)	162 (92.6)	
Bacterial pneumonia	99 (17.0)	69 (17.0)	30 (17.1)	
Viral pneumonia	312 (53.6)	229 (56.3)	83 (47.4)	
Other pneumonia	133 (22.9)	84 (20.6)	49 (28.0)	
Extrapulmonary	38 (6.5)	25 (6.1)	13 (7.4)	
SOFA score at ECMO initiation	10.0 (7.0–13.0)	9.0 (7.0–12.0)	12.0 (8.0–14.0)	< 0.001
P/F ratio before ECMO initiation^b^	89.1 ± 37.0	89.3 ± 37.9	88.6 ± 34.9	0.82
pH before ECMO initiation^c^	7.30 ± 0.13	7.30 ± 0.13	7.29 ± 0.14	0.41
Mechanical ventilation settings before ECMO initiation				
Mechanical ventilator mode				0.76
Pressure control, *n* (%)	534 (91.8)	376 (92.4)	158 (90.3)	
Volume control, *n* (%)	33 (5.7)	21 (5.2)	12 (6.9)	
PEEP, cmH_2_O^d^	12.1 ± 3.9	12.1 ± 3.9	12.0 ± 3.9	0.87
Dynamic driving pressure, cmH_2_O^e^	16.5 ± 6.9	16.4 ± 6.9	16.7 ± 7.0	0.68
Murray Lung Injury Score^f^	3.25 (2.75–3.50)	3.25 (2.75–3.50)	3.25 (2.75–3.50)	0.19
Static lung compliance, mL/cmH_2_O^g^	29.2 ± 15.9	29.4 ± 16.2	28.9 ± 15.4	0.76
Use of neuromuscular blockers before ECMO support, *n* (%)	242 (41.6)	165 (40.5)	77 (44.0)	0.44
Prone positioning before ECMO support, *n* (%)	94 (16.2)	64 (15.7)	30 (17.1)	0.67

Data are presented as the median and interquartile range (25–75% percentile), mean ± standard deviation, or absolute frequency with percentage

ARDS: acute respiratory distress syndrome; BMI: body mass index; ECMO: extracorporeal membrane oxygenation; MV: mechanical ventilation; SOFA: Sequential Organ Failure Assessment; P/F: partial pressure of oxygen/fraction of inspired oxygen ratio; PEEP: positive end-expiratory pressure

^a^Missing values = 4

^b^Missing values = 16

^c^Missing values = 18

^d^Missing values = 19

^e^Missing values = 95

^f^Missing values = 8

^g^Missing values = 129

**Table 2:** Baseline characteristics of all participants after V-V ECMO instauration

	All patients (*n* = 582)	Survived (*n* = 407)	Died (*n* = 175)	*p***-**value
*Mechanical ventilation settings during ECMO support*				
Mechanical ventilator mode				0.90
Pressure control, *n* (%)	557 (95.9)	391 (96.1)	166 (95.4)	
Volume control, *n* (%)	13 (2.2)	9 (2.2)	4 (2.3)	
PEEP during 1–3 days, cmH_2_O^a^	10.0 ± 3.4	10.1 ± 3.4	9.7 ± 3.3	0.24
Dynamic driving pressure during 1–3 days, cmH_2_O^b^	10.0 ± 5.2	10.3 ± 5.5	9.5 ± 4.4	0.06
PEEP during 4–7 days, cmH_2_O^c^	10.3 ± 5.2	10.4 ± 5.9	9.9 ± 3.0	0.23
Dynamic driving pressure during 4–7 days, cmH_2_O^d^	10.0 ± 5.0	10.4 ± 5.4	9.2 ± 3.8	0.016
ECMO setting				
Blood flow, L/min^e^	3.9 ± 0.7	3.9 ± 0.7	3.9 ± 0.7	0.28
Sweep gas, L/min^f^	3.7 ± 1.9	3.8 ± 1.9	3.7 ± 2.0	0.84
FdO2	1.0 (1.0–1.0)	1.0 (1.0–1.0)	1.0 (1.0–1.0)	> 0.99
Cannulation site (drainage-return cannula)				0.30
Jugular–femoral, *n* (%)	315 (54.1)	229 (56.3)	86 (49.1)	
Femoral–jugular, *n* (%)	236 (40.6)	155 (38.1)	81 (46.3)	
Femoral–femoral, *n* (%)	25 (4.3)	19 (4.7)	6 (3.4)	
*Treatment during ECMO support*				
Corticosteroid use within 2 weeks, *n* (%)^g^	399 (68.7)	269 (66.1)	130 (74.7)	0.038
Neuromuscular blocker use within 48 h, *n* (%)^h^	325 (56.1)	231 (56.9)	94 (54.3)	0.59
Prone positioning within 2 weeks, *n* (%)^i^	147 (25.4)	102 (25.2)	45 (26.0)	0.83
Duration of ECMO run, days^j^	11.0 (7.0–19.0)	9.5 (7.0–14.0)	19.0 (9.0–36.0)	< 0.001
Re-cannulation of ECMO, *n* (%)^k^	22 (3.8)	11 (2.7)	11 (6.3)	0.046

Data are presented as the median and interquartile range (25–75% percentile), mean ± standard deviation, or absolute frequency with percentage

ECMO: extracorporeal membrane oxygenation; FdO2: fraction of oxygen delivered from the blender; PEEP: positive end-expiratory pressure

^a^Missing values = 4

^b^Missing values = 16

^c^Missing values = 34

^d^Missing values = 44

^e^Missing values = 5

^f^Missing values = 8

^g^Missing value = 1

^h^Missing values = 2

^i^Missing values = 4

^j^Missing values = 5

^k^Missing values = 2

**Fig. 2:**
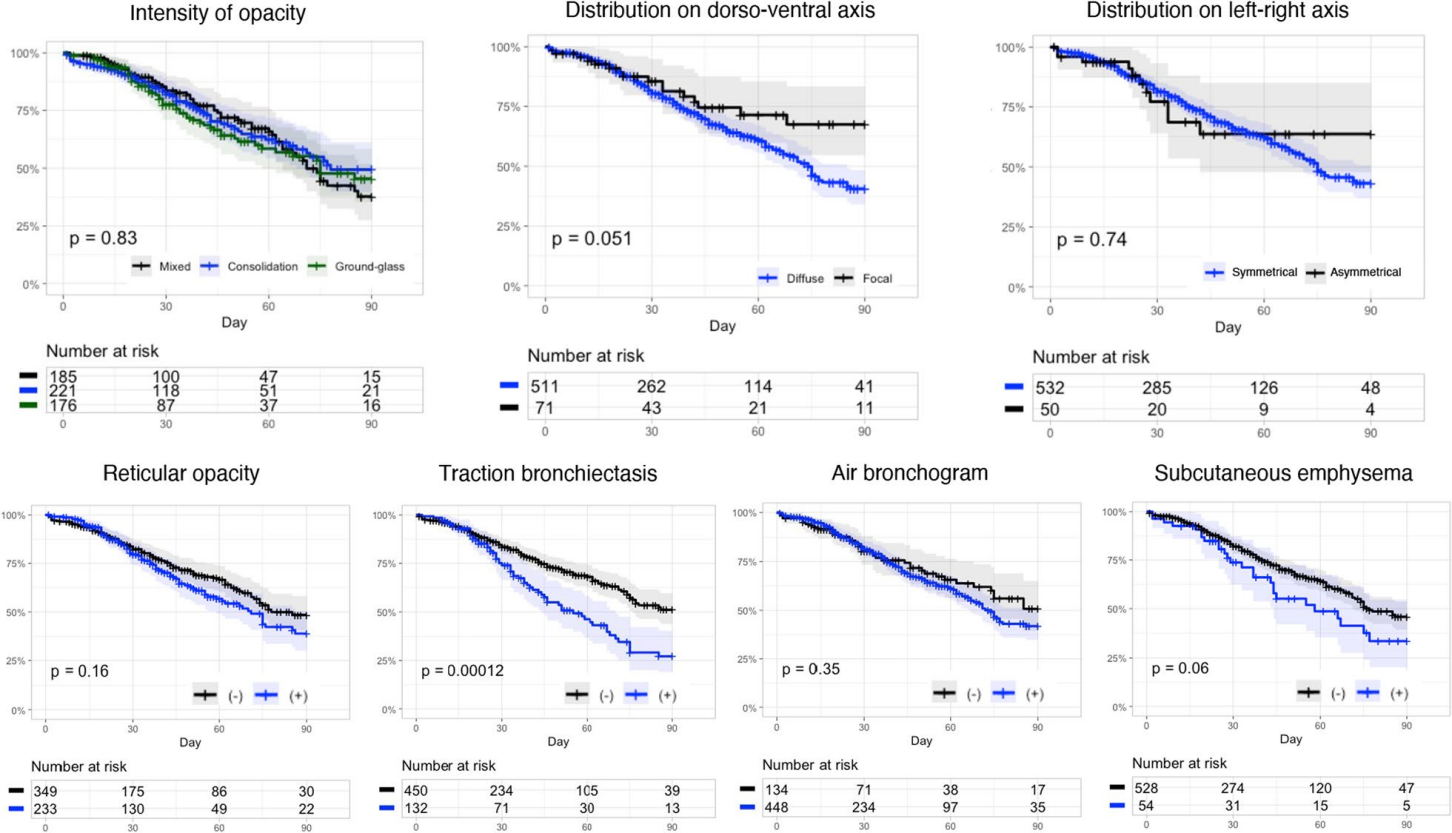
Survival curve for each chest computed tomography finding related to pulmonary opacity and subcutaneous emphysema. Log-rank tests were performed for the survival analysis

The interpretations of the chest CT findings of the analyzed patients are summarized in Additional file 1: Table S3. The interpretations of the chest CT findings analyzed according to the duration between the start of MV and V-V ECMO support initiation and primary reasons for ARDS are shown in Additional file 1: Fig. S4. We plotted Kaplan–Meier curves for the findings related to pulmonary opacity and subcutaneous emphysema (Fig. 2). The curves for the other findings are shown in Additional file 1: Fig. S5. The 90-day in-hospital mortality was significantly higher in patients with traction bronchiectasis than in those without (log-rank test: *p* < 0.001); however, no statistical significance was observed in the associations of other CT interpretations. The multivariate Cox regression analysis showed that the presence of traction bronchiectasis or subcutaneous emphysema increased the risk of 90-day in-hospital mortality (traction bronchiectasis; hazard ratio [HR] 1.77 [95% confidence interval {CI} 1.19–2.63], *p* = 0.005, subcutaneous emphysema; HR 1.97 [1.02–3.79], *p* = 0.044; see Additional file 1: Table S4).

**Fig. 3:**
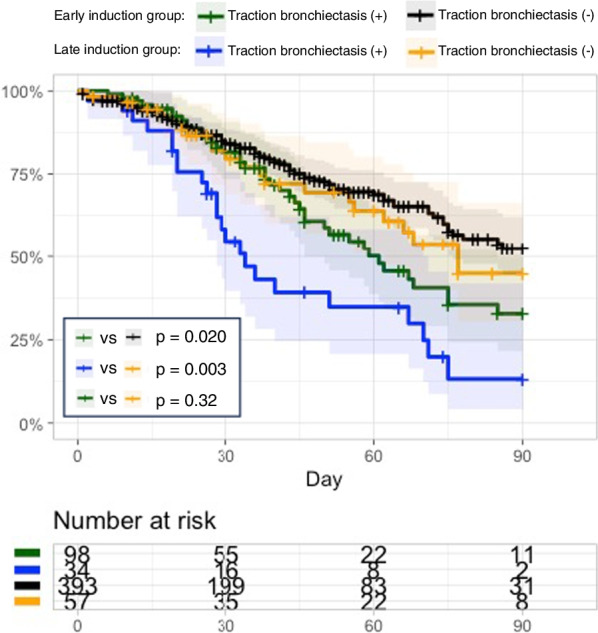
Survival curves of the patients with and without traction bronchiectasis. Log-rank tests were performed for the survival analysis, and curves were stratified according to early or late induction of extracorporeal membrane oxygenation

For the subgroup analyses, we stratified all analyzed patients according to whether the duration between the start of MV and V-V ECMO support initiation was ≥ 7 days or not (early vs. late induction group). The 90-day in-hospital mortality was 39.8% (39/98) and 23.4% (92/393) in the early induction groups with and without traction bronchiectasis, respectively, and 67.7% (23/34) and 36.8% (21/57) in the late induction groups with and without traction bronchiectasis, respectively. The log-rank test showed that mortality was greater in patients with traction bronchiectasis than in those without, regardless of early or late induction (early, *p* = 0.020; late, *p* = 0.003). There was no significant difference between the patients with traction bronchiectasis in the early induction group and those without traction bronchiectasis in the late induction group (*p* = 0.32) (Fig. 3). Kaplan–Meier curves of traction bronchiectasis according to the primary reason for ARDS (excluding patients with extrapulmonary reasons as the primary cause of ARDS because there were only three with traction bronchiectasis on their chest CT scans) were also plotted. A trend was observed where the proportion of patients with traction bronchiectasis was greater than that of those without the sign, regardless of the primary reason for ARDS (see Additional file 1: Fig. S6). The presence of traction bronchiectasis also significantly decreased the odds ratio for successful liberation from V-V ECMO in the multivariate logistic regression analysis (odds ratio: 0.27 [95% CI 0.14–0.52], *p* < 0.001; see Additional file 1: Table S5).

**Fig. 4:**
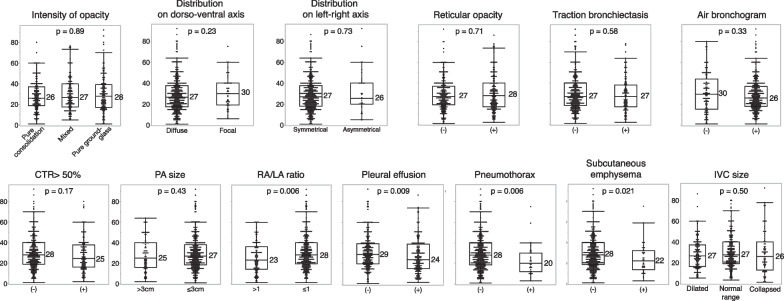
Association between decreased value of static lung compliance and each chest computed tomography finding. Numbers close to the box plot represent the median values. The Mann–Whitney *U* test was performed. CTR: cardiothoracic ratio; IVC: inferior vena cava; LA: left atrium; PA: pulmonary artery; RA: right atrium

In addition, for 453 patients for whom the values of static lung compliance were available, we evaluated the association between the values and each CT finding at the start of V-V ECMO support. Decreased values of static lung compliance were associated with the presence of pleural effusion, pneumothorax, and subcutaneous emphysema and a right atrium/left atrium ratio of > 1, although there were no findings related to the intensity of opacity, distribution of opacity, or fibroproliferative changes (Fig. 4).

## Discussion

In this study, we reported the profile of the J-CARVE registry, which is a large multicenter database of patients with severe ARDS on V-V ECMO support, including chest CT imaging data at the initiation of V-V ECMO support. This registry consists of data from 24 institutes across Japan, which has the highest number of CT scanners among the Organization for Economic Cooperation and Development countries and where CT examinations are much easier to access than those in other countries [20]. In fact, our database included the chest CT imaging data at the initiation of V-V ECMO support in most of the registered patients (> 80%).

Our registry is the first to report a large sample size from Japan to describe the outcomes of patients with severe ARDS on V-V ECMO. According to our data, > 80% of all patients were successfully liberated from V-V ECMO, and their ICU mortality was approximately 30%, which is comparable to that of 25–35% reported in previous studies, including an international report by Schmidt et al. [10, 15, 21]. The SOFA scores between our study and Schmidt et al.’s study were also comparable (9.8 ± 4.1 vs 10.4 ± 4.0), which means that the severity may be similar between the two studies. As for the treatment before and after V-V ECMO, the percentage of patients who underwent neuromuscular blocker therapy (56%) or prone positioning (26%) during V-V ECMO was higher than that reported in Schmidt et al.’s study (41% and 6%, respectively); in contrast, the percentage of patients who underwent these therapies before V-V ECMO was lower (our registry: 42% and 16%; Schmidt et al.’s study: 62% and 26%, respectively). This may be due to the difference in general treatment strategies between Japan, where a V-V ECMO is initiated without any attempt of other treatments, and other countries. We are interested in performing a future study to evaluate their outcomes after adjusting for the content of treatments before and after V-V ECMO between Japan and other countries.

Our findings highlight the importance of identifying traction bronchiectasis when evaluating the risk of mortality as well as predicting successful liberation in patients with severe ARDS on V-V ECMO, which aligns with previous findings targeting general populations of patients with ARDS without V-V ECMO [22–24]. Although our previous retrospective cohort study failed to show a statistically significant association between traction bronchiectasis and hospital mortality, this was possibly due to a small sample size [25]. Traction bronchiectasis is a reliable index of the degree of fibroproliferation in the pathophysiology of ARDS, especially ARDS with a diffuse alveolar damage pattern pathologically, and it was reported to be linked to the need for prolonged mechanical ventilatory support and worse outcomes [26]. Pathophysiologically, the presence of fibroproliferative changes may represent more severe lung injury like diffuse alveolar damage because ARDS is a complex syndrome with diverse pathological manifestations [27].

In our study, the mortality of patients in the late induction group was similar to that of those in the early induction group if traction bronchiectasis was absent. International guidelines suggest that the indication for V-V ECMO is limited to patients who receive MV within 7 days, based on previous studies showing the possibility that lung injuries in patients receiving MV for > 7 days are irreversible, leading to a decreased survival rate [21, 28]. However, our data suggest it is the existence of traction bronchiectasis, rather than the timing alone, that determines their survival, although the difference in survival during the short periods after V-V ECMO support has to be carefully evaluated in a future study because some survival curves in our results showed intersection during short periods.

In patients with severe ARDS, all of whom should have decreased static lung compliance, the values of static compliance were not associated with any findings related to pulmonary opacity, including intensity, distribution, and fibroproliferative changes. A previous study showed that static compliance was correlated with the amount of normally aerated lung tissues, and not with poorly aerated or nonaerated tissues [29], suggesting that the static compliance does not reflect the pathophysiology of severe ARDS, in which many parts of the lung are poorly aerated or nonaerated. Notably, the values of static compliance were similar between the patients who survived and those who died in our study (29.4 vs. 28.9 mL/cmH_2_O), which is consistent with the results of several studies reporting that static lung compliance is not associated with mortality [15]. We believe that our results support the importance of evaluation using other indices such as the presence of traction bronchiectasis, which cannot be assessed using respiratory mechanics.

In contrast, chest CT findings related to changes outside of pulmonary opacity, such as the presence of pneumothorax and subcutaneous emphysema, were significantly associated with a decreased value of static lung compliance. In particular, the presence of subcutaneous emphysema is the main macroscopic sign of barotrauma, which worsens the outcomes in patients with ARDS [30, 31]. Our data showed a significant association between the presence of subcutaneous emphysema and increased mortality, which is consistent with these previous findings.

Our study has some limitations. First, although all participating hospitals followed the guidelines for the indication for V-V ECMO, the final decision was made according to the preference of each participating hospital. Second, the values of positive end-expiratory pressure (PEEP) at the time of the chest CT examination were varied. PEEP-induced alveolar recruitment can transform poorly aerated lung areas into normally aerated lung areas [32]. Nevertheless, we believe that the interpretation of the presence of traction bronchiectasis and subcutaneous emphysema is unlikely to be significantly influenced by PEEP, because the values of PEEP during chest CT were similar between those with and without these findings (traction bronchiectasis (+) vs (−); 10.3 ± 4.4 cmH_2_O vs 10.7 ± 4.9 cmH_2_O, and subcutaneous emphysema (+) vs (−); 9.6 ± 3.3 cmH_2_O vs 10.7 ± 4.9 cmH_2_O). However, this does not mean that the continuous setting for MV until CT examination (not just timing at CT examination) does not influence the presence of any chest CT findings. Third, we cannot exclude a potential immortal time bias, although the 90-day survival rate was not markedly different between patients who underwent chest CT examination and those who did not (69.4% [455/656] vs 63.4% [26/41]). Fourth, several details regarding the measurements of some respiratory mechanics variables including static lung compliance at each participating hospital were unknown, which is a common limitation in large multicenter retrospective studies. Fifth, we cannot completely exclude the possibility that our database included the data of some patients with ARDS mimics, although we believe that our study is still worthwhile, as our registry contains real-world data. Finally, lung transplantation is rarely performed in Japan (none of the patients in this study underwent transplantation), which may have affected the indications and withdrawal of V-V ECMO support.

## Conclusions

Traction bronchiectasis and subcutaneous emphysema increased the risk of 90-day in-hospital mortality in patients with severe ARDS who required V-V ECMO.

### Supplementary Information

**Additional file 1: **SMethods. **Figure S1.** Representative images of each of the characteristic pulmonary opacities on chest computed tomography scans. **Figure S2.** Distribution of registered patients by years. **Figure S3.** Cumulative proportion of the duration (h) between chest computed tomography examinations and initiation of veno-venous extracorporeal membrane oxygenation support. **Figure S4.** Characteristics of the chest computed tomography findings according to the mechanical ventilation–extracorporeal membrane oxygenation support duration and the underlying etiology of the acute respiratory distress syndrome. **Figure S5.** Survival curve of the chest computed tomography findings related to changes outside of the pulmonary opacity (excluding subcutaneous emphysema). **Figure S6**. Survival curve of participants with and without traction bronchiectasis separately according to the underlying etiology of acute respiratory distress syndrome. **Table S1.** Concordance rates between two evaluators. **Table S2.** Basic information of the participating hospitals. **Table S3**. Characteristics of chest computed tomography findings. **Table S4.** Results of multivariate Cox regression analysis of the relationship between V-V ECMO support initiation and 90-day in-hospital mortality. **Table S5.** Results of multivariate logistic regression analysis for successful ECMO liberation.

## Data Availability

The datasets used and analyzed during the current study are available from the corresponding author upon reasonable request.

## Abbreviations

ARDS: Acute respiratory distress syndrome
CI: Confidence interval
CT: Computed tomography
HR: Hazard ratio
ICUs: Intensive care units
J-CARVE registry: Japan Chest CT for ARDS requiring V-V ECMO registry
MV: Mechanical ventilation
PEEP: Positive end-expiratory pressure
P/F ratio: PaO2/FiO2 ratio
UMIN-CTR: University Hospital Medical Information Network Clinical Trials Registry
V-V ECMO: Veno-venous extracorporeal membrane oxygenation
