# Supplementary material for: Chest CT findings in severe acute respiratory distress syndrome requiring V-V ECMO: J-CARVE registry
Source: J Intensive Care. 2024 Jan 26;12:5. doi: 10.1186/s40560-023-00715-x (PMC10811928; doi:10.1186/s40560-023-00715-x)
Supplement: Supplementary file 1 — Additional file 1: SMethods. Figure S1. Representative images of each of the characteristic pulmonary opacities on chest computed tomography scans. Figure S2. Distribution of registered patients by years. Figure S3. Cumulative proportion of the duration (h) between chest computed tomography examinations and initiation of veno-venous extracorporeal membrane oxygenation support. Figure S4. Characteristics of the chest computed tomography findings according to the mechanical ventilation–extracorporeal membrane oxygenation support duration and the underlying etiology of the acute respiratory distress syndrome. Figure S5. Survival curve of the chest computed tomography findings related to changes outside of the pulmonary opacity (excluding subcutaneous emphysema). Figure S6. Survival curve of participants with and without traction bronchiectasis separately according to the underlying etiology of acute respiratory distress syndrome. Table S1. Concordance rates between two evaluators. Table S2. Basic information of the participating hospitals. Table S3. Characteristics of chest computed tomography findings. Table S4. Results of multivariate Cox regression analysis of the relationship between V-V ECMO support initiation and 90-day in-hospital mortality. Table S5. Results of multivariate logistic regression analysis for successful ECMO liberation. [file 40560_2023_715_MOESM1_ESM.docx]

**Additional files**

**Chest CT findings in severe acute respiratory distress syndrome needing V-V ECMO: J-CARVE registry**

Mitsuaki Nishikimi^1,^ *; Shinichiro Ohshimo^1^; Wataru Fukumoto^2^; Jun Hamaguchi^3^; Kazuki Matsumura^3^; Kenji Fujizuka^4^; Yoshihiro Hagiwara^5^; Ryuichi Nakayama^6^; Naofumi Bunya^6^; Junichi Maruyama^7^; Toshikazu Abe^8^; Tatsuhiko Anzai^9^; Yoshitaka Ogata^10^; Hiromichi Naito^11^; Yu Amemiya^12^; Tokuji Ikeda^13^; Masayuki Yagi^14^; Yutaro Furukawa^15^; Hayato Taniguchi^16^; Tsukasa Yagi^17^; Ken Katsuta^18^; Daisuke Konno^19^; Ginga Suzuki^20^; Yuki Kawasaki^21^; Noriyuki Hattori^22^; Tomoyuki Nakamura^23^; Natsuki Kondo^24^; Hitoshi Kikuchi^25^; Shinichi Kai^26^; Kazuo Awai^2^; Kunihiko Takahashi^9^; Nobuaki Shime^1^; J-CARVE registry group.

J-CARVE registry group authors: Junki Ishii^1^; Takayuki Ogura^5^; Mitsunobu Nakamura^4^; Keiki Shimizu^3^; Tatsutoshi Shimatani^1^; Mamoru Masuda^4^.

**SMethods**

**Data collection and quality control**

The J-CARVE registry includes data on basic demographics and comorbidities, laboratory tests, settings of mechanical ventilation (MV) and the measured values (before, during, and after ECMO support), treatments, outcomes, and anonymized chest CT scans at the time closest to the initiation of ECMO support. Even if registered patients were transferred to a referral hospital after the start of MV in the primary hospital, the data on the day that MV was initiated in the primary hospital were collected. Physicians or medical staff, in cooperation with physicians, entered the anonymized data into the electronic data capture (EDC) database (available from https://www.ace-registry.net) during the registry period. As in a previous study, if unspecified, the peak inspiratory pressure was considered to be the same as the plateau pressure in pressure-regulated modes [1]. Dynamic driving pressure was calculated as peak inspiratory pressure minus positive end-expiratory pressure according to previous studies [2, 3]. Static lung compliance was calculated using the formula: (tidal volume)/(plateau pressure - positive end-expiratory pressure). Data were logically checked using the system and finally confirmed by the J-CARVE registry committee, including intensive care unit and emergency physicians who had sufficient experience in the management of V-V ECMO, epidemiologists, and statisticians. If the data form was incomplete, the committee member returned it to the respective institution, and the data were completed as much as possible.

Anonymized chest CT data were uploaded to the EDC during the registry-creation period. Axial slice (5 mm slice thickness) images were obtained at the closest timepoint from the start of V-V ECMO support. If multiple CT scans were conducted, only the chest CT scan at the closest time to the start of V-V ECMO support was uploaded. The uploaded files were mainly in portable network graphics, joint photographic experts group, or digital imaging and communications in medicine formats. If the CT image included the abdominal and pelvic regions or if the thin-slice scan was simultaneously obtained, all of the images were uploaded.

**References**

1. Schmidt M, Pham T, Arcadipane A, Agerstrand C, Ohshimo S, Pellegrino V, et al. Mechanical ventilation management during extracorporeal membrane oxygenation for acute respiratory distress syndrome. An international multicenter prospective cohort. Am J Respir Crit Care Med. 2019;200:1002-12.

2. Urner M, Jüni P, Hansen B, Wettstein MS, Ferguson ND, Fan E. Time-varying intensity of mechanical ventilation and mortality in patients with acute respiratory failure: a registry-based, prospective cohort study. Lancet Respir Med. 2020;8:905-13.

3. Douville NJ, McMurry TL, Ma JZ, Naik BI, Mathis MR, Colquhoun D, et al. Airway driving pressure is associated with postoperative pulmonary complications after major abdominal surgery: a multicentre retrospective observational cohort study. BJA Open. 2022;4:100099.

1. Schmidt M, Pham T, Arcadipane A, Agerstrand C, Ohshimo S, Pellegrino V, Vuylsteke A, Guervilly C, McGuinness S, Pierard S, Breeding J, Stewart C, Ching SSW, Camuso JM, Stephens RS, King B, Herr D, Schultz MJ, Neuville M, Zogheib E, Mira JP, Roze H, Pierrot M, Tobin A, Hodgson C, Chevret S, Brodie D, Combes A, (2019) Mechanical Ventilation Management during Extracorporeal Membrane Oxygenation for Acute Respiratory Distress Syndrome. An International Multicenter Prospective Cohort. Am J Respir Crit Care Med 200: 1002-1012

2. Urner M, Juni P, Hansen B, Wettstein MS, Ferguson ND, Fan E, (2020) Time-varying intensity of mechanical ventilation and mortality in patients with acute respiratory failure: a registry-based, prospective cohort study. Lancet Respir Med 8: 905-913

3. Douville NJ, McMurry TL, Ma JZ, Naik BI, Mathis MR, Colquhoun D, Kheterpal S, Pace NL, Hedrick TL, Blank RS, Multicenter Perioperative Outcomes Group Perioperative Clinical Research C, (2022) Airway driving pressure is associated with postoperative pulmonary complications after major abdominal surgery: a multicentre retrospective observational cohort study. BJA Open 4

**Figure S1.** **Representative images of each of the characteristic pulmonary opacities on chest computed tomography scans**

**Figure S2. Distribution of registered patients by years**

**Figure S3.** **Cumulative proportion of the duration (h) between chest computed tomography examinations and initiation of veno-venous extracorporeal membrane oxygenation support**

**Figure S4.** **Characteristics of the chest computed tomography findings according to the mechanical ventilation–extracorporeal membrane oxygenation support duration and the underlying etiology of the acute respiratory distress syndrome**

The percentage of patients who had each finding is presented according to the duration between mechanical ventilation and ECMO support (early [n=491] and late [n=91] induction groups) and underlying etiology of acute respiratory distress syndrome (bacterial pneumonia [n=99], viral pneumonia [n=312], other pneumonia [n=133], and extrapulmonary reason [n=38]).

Abbreviations: Bacterial, bacterial pneumonia; CTR, cardiothoracic ratio; Early, early induction group; Extrapulmonary, extrapulmonary cause; IVC, inferior vena cava; LA, left atrium; Late, late induction group; Others, other pneumonia; PA, pulmonary artery; RA, right atrium; Virus, viral pneumonia.

**Figure S5. Survival curve of the chest computed tomography findings related to changes outside of the pulmonary opacity (excluding subcutaneous emphysema)**

Log-rank tests were performed for the survival analysis. CTR, cardiothoracic ratio; IVC, inferior vena cava; LA, left atrium; PA, pulmonary artery; RA, right atrium.

**Figure S6. Survival curve of participants with and without traction bronchiectasis separately according to the underlying etiology of acute respiratory distress syndrome**

Log rank tests were performed for the survival analysis.

**Table S1. Concordance rates between two evaluators**

|  | **Concordance rate (%)** |
| --- | --- |
| Intensity of opacity | 75.9 |
| Distribution of opacity | |
| Distribution on dorso–ventral axis, diffuse (vs. focal) | 90.5 |
| Distribution on left–right axis, symmetrical (vs. asymmetrical) | 92.4 |
| Opacity in each lobe >50% | |
| Right upper | 87.5 |
| Right middle | 80.9 |
| Right lower | 92.6 |
| Left upper | 84.4 |
| Left lower | 90.9 |
| Degree of fibroproliferation of opacity | |
| Reticular opacity | 74.7 |
| Traction bronchiectasis | 85.1 |
| Others | |
| Air bronchogram | 83.5 |
| CTR >50% | 81.3 |
| PA size >3 cm | 83.3 |
| RA/LA ratio >1 | 85.6 |
| Pleural effusion | 82.0 |
| Pneumothorax | 97.9 |
| Subcutaneous emphysema | 96.6 |
| IVC size | 76.5 |

Abbreviations: CTR, cardiothoracic ratio; IVC, inferior vena cava; LA, left atrium; PA, pulmonary artery; RA, right atrium.

**Table S2. Basic information of the participating hospitals**

|  | **All hospitals (n=24)** |
| --- | --- |
| Hospital type, academic, n (%) | 14 (58.3) |
| Bed capacity, beds |  |
| Total | 710 (605–920) |
| Intensive care unit | 18 (9–22) |
| Number of the patients with V-V ECMO treated in the last year | 10 (4–12) |
| Type of ICU, n (%) |  |
| Closed system | 7 (29.2) |
| Semi-closed system | 14 (58.3) |
| Opened system | 3 (12.5) |
| Most usual site for cannulation (Drainage-return cannula), n (%) |  |
| Femoral–jugular | 8 (33.3) |
| Jugular–femoral | 16 (66.7) |
| Most usual respirator mode |  |
| Pressure control | 23 (95.8) |
| Volume control | 1 (4.2) |
| Limited tidal volume during V-V ECMO support, n (%) |  |
| Usual lung protective care (≤6 mL/IBW kg) | 13 (54.2) |
| Ultra-lung protective care (≤3 mL/IBW kg) | 11 (45.8) |
| Most usual values of PEEP setting (cmH_2_O), n (%) |  |
| <5 | 0 (0.0) |
| 5–8 | 7 (29.2) |
| 9–11 | 8 (33.3) |
| 12–15 | 2 (8.3) |
| >15 | 0 (0.0) |
| No setting value | 7 (29.2) |

Data are presented as the median and interquartile range (25–75% percentile) or absolute frequency with percentage.

Abbreviations: IBW, ideal body weight; ICU, intensive care unit; PEEP, positive end-expiratory pressure; V-V ECMO, veno-venous extracorporeal membrane oxygenation.

**Table S3. Characteristics of chest computed tomography findings**

|  | **All**  **n=582** | **Survived**  **n=407** | **Died**  **n=175** | ***P*-value** |
| --- | --- | --- | --- | --- |
| Intensity of opacity |  |  |  | 0.69 |
| Pure consolidation, *n* (%) | 221 (38.0) | 159 (39.1) | 62 (35.4) |  |
| Mixed, *n* (%) | 185 (31.8) | 128 (31.5) | 57 (32.6) |  |
| Pure ground-glass, *n* (%) | 176 (30.2) | 120 (29.5) | 56 (32.0) |  |
| Distribution of opacity | | | | |
| Distribution on the dorso–ventral axis, diffuse (vs. focal), *n* (%) | 511 (87.8) | 352 (86.5) | 159 (90.9) | 0.13 |
| Distribution on the left–right axis, symmetrical (vs. asymmetrical), *n* (%) | 532 (91.4) | 368 (90.4) | 164 (93.7) | 0.19 |
| Opacity in each lobe >50% | | | | |
| Right upper, *n* (%) | 437 (75.1) | 297 (73.0) | 140 (80.0) | 0.07 |
| Right middle, *n* (%) | 398 (68.4) | 271 (66.6) | 127 (72.6) | 0.15 |
| Right lower, *n* (%) | 519 (89.2) | 364 (89.4) | 155 (88.6) | 0.76 |
| Left upper, *n* (%) | 416 (71.5) | 278 (68.3) | 138 (78.9) | 0.009 |
| Left lower, *n* (%) | 520 (89.4) | 359 (88.2) | 161 (92.0) | 0.16 |
| Degree of fibroproliferation of opacity | | | | |
| Reticular opacity, *n* (%) | 233 (40.0) | 152 (37.4) | 81 (46.3) | 0.044 |
| Traction bronchiectasis, *n* (%) | 132 (22.7) | 70 (17.2) | 62 (35.4) | <0.001 |
| Others | | | | |
| Air bronchogram, *n* (%) | 448 (77.0) | 313 (76.9) | 135 (77.1) | 0.95 |
| CTR >50%, *n* (%) | 176 (30.2) | 117 (28.8) | 59 (33.7) | 0.23 |
| PA size >3 cm, *n* (%) | 487 (83.7) | 336 (82.6) | 151 (86.3) | 0.26 |
| RA/LA ratio >1, *n* (%) | 455 (78.2) | 321 (78.9) | 134 (76.6) | 0.54 |
| Pleural effusion, *n* (%) | 238 (40.9) | 158 (38.8) | 80 (45.7) | 0.12 |
| Pneumothorax, *n* (%) | 41 (7.0) | 24 (5.9) | 17 (9.7) | 0.11 |
| Subcutaneous emphysema, *n* (%) | 54 (9.3) | 28 (6.9) | 26 (14.9) | 0.003 |
| IVC size |  |  |  | 0.08 |
| Dilated (≥21 mm), *n* (%) | 174 (29.9) | 122 (30.0) | 52 (29.7) |  |
| Collapsed (≤7 mm), *n* (%) | 87 (15.0) | 52 (12.8) | 35 (20.0) |  |

Abbreviations: CTR, cardiothoracic ratio; IVC; inferior vena cava; LA, left atrium; PA, pulmonary artery; RA, right atrium.

**Table S4. Results of multivariate Cox regression analysis of the relationship between V-V ECMO support initiation and 90-day in-hospital mortality**

|  | **Adjusted HR (95% CI)** | ***P*-value** |
| --- | --- | --- |
| Age, years | 1.02 (1.00–1.04) | 0.001 |
| Sex, male | 0.74 (0.52–1.06) | 0.10 |
| BMI | 1.03 (0.99–1.06) | 0.11 |
| Hypertension | 0.82 (0.59–1.15) | 0.25 |
| Diabetes | 0.79 (0.54–1.16) | 0.23 |
| Chronic kidney disease | 0.82 (0.46–1.47) | 0.51 |
| Obstructive lung disease | 1.08 (0.69–1.70) | 0.74 |
| Interstitial lung disease | 1.22 (0.59–2.54) | 0.59 |
| Chronic heart failure | 1.85 (0.96–3.57) | 0.07 |
| Duration between MV and ECMO instauration | 1.02 (0.99–1.05) | 0.11 |
| Primary reason for ARDS (ref: Extrapulmonary) |  |  |
| Bacterial pneumonia | 0.81 (0.39–1.67) | 0.57 |
| Viral pneumonia | 0.87 (0.44–1.72) | 0.68 |
| Other pneumonia | 1.05 (0.54–2.06) | 0.88 |
| SOFA score at ECMO instauration | 1.07 (1.02–1.12) | 0.003 |
| Muscle relaxants used before ECMO support | 0.93 (0.67–1.28) | 0.64 |
| Prone positioning before ECMO support | 1.01 (0.64–1.60) | 0.96 |
| Chest CT findings |  |  |
| Intensity of opacity (Ref: Pure consolidation) |  |  |
| Mixed | 0.81 (0.52–1.26) | 0.34 |
| Pure ground-glass | 0.97 (0.58–1.60) | 0.89 |
| Distribution on the dorso–ventral axis, diffuse (Ref: focal) | 1.84 (1.00–3.39) | 0.05 |
| Distribution on the left–right axis, symmetrical (Ref:  asymmetrical) | 1.04 (0.55–1.98) | 0.91 |
| Reticular opacity | 1.00 (0.69–1.46) | >0.99 |
| Traction bronchiectasis | 1.77 (1.19–2.63) | 0.005 |
| Air bronchogram | 1.40 (0.93–2.11) | 0.11 |
| CTR >50% | 1.17 (0.82–1.68) | 0.38 |
| PA size >3 cm | 0.65 (0.40–1.07) | 0.09 |
| RA/LA ratio >1 | 0.68 (0.45–1.05) | 0.08 |
| Pleural effusion | 0.88 (0.61–1.25) | 0.46 |
| Pneumothorax | 0.89 (0.39–2.02) | 0.78 |
| Subcutaneous emphysema | 1.97 (1.02–3.79) | 0.044 |
| IVC size (Ref: Normal range [7–21 mm]) |  |  |
| Dilated (≥21 mm) | 1.00 (0.69–1.47) | 0.98 |
| Collapsed (≤7 mm) | 1.19 (0.77–1.82) | 0.44 |

Abbreviations: ARDS, acute respiratory distress syndrome; BMI, body mass index; CTR, cardiothoracic ratio; ECMO, extracorporeal membrane oxygenation; HR, hazard ratio; IVC, inferior vena cava; LA, left atrium; MV, mechanical ventilation; PA, pulmonary artery; P/F ratio, partial pressure of oxygen/fraction of inspired oxygen ratio; RA, right atrium; SOFA, Sequential Organ Failure Assessment.

**Table S5. Results of multivariate logistic regression analysis for successful ECMO liberation**

|  | **Adjusted OR (95% CI)** | ***P*-value** |
| --- | --- | --- |
| Age, years | 0.96 (0.93–0.98) | <0.001 |
| Sex, male | 0.97 (0.55–1.74) | 0.93 |
| BMI | 0.99 (0.94–1.04) | 0.77 |
| Hypertension | 0.97 (0.57–1.64) | 0.92 |
| Diabetes | 1.98 (1.10–3.59) | 0.024 |
| Chronic kidney disease | 1.70 (0.69–4.20) | 0.25 |
| Obstructive lung disease | 0.64 (0.33–1.24) | 0.19 |
| Interstitial lung disease | 2.29 (0.65–8.04) | 0.20 |
| Chronic heart failure | 0.85 (0.33–2.20) | 0.74 |
| Duration between MV and ECMO instauration | 0.94 (0.89–0.99) | 0.013 |
| Primary reason for ARDS (ref: Extrapulmonary) |  |  |
| Bacterial pneumonia | 0.99 (0.39–1.67) | 0.57 |
| Viral pneumonia | 0.93 (0.31–2.79) | 0.90 |
| Other pneumonia | 0.77 (0.25–2.31) | 0.64 |
| SOFA score at ECMO instauration | 0.92 (0.86–0.99) | 0.022 |
| Muscle relaxants used before ECMO support | 0.95 (0.57–1.58) | 0.83 |
| Prone positioning before ECMO support | 0.68 (0.34–1.35) | 0.27 |
| Chest CT findings |  |  |
| Intensity of opacity (Ref: Pure consolidation) |  |  |
| Mixed | 1.59 (0.78–3.25) | 0.20 |
| Pure ground-glass | 1.15 (0.51–2.59) | 0.73 |
| Distribution on the dorso–ventral axis, diffuse (Ref: focal) | 0.77 (0.33–1.81) | 0.55 |
| Distribution on the left–right axis, symmetrical (Ref:  asymmetrical) | 0.87 (0.35–2.13) | 0.75 |
| Reticular opacity | 1.44 (0.79–2.60) | 0.23 |
| Traction bronchiectasis | 0.27 (0.14–0.52) | <0.001 |
| Air bronchogram | 0.83 (0.44–1.55) | 0.55 |
| CTR >50% | 0.41 (0.24–0.72) | 0.002 |
| PA size >3 cm | 1.32 (0.64–2.74) | 0.45 |
| RA/LA ratio >1 | 1.91 (0.97–3.75) | 0.06 |
| Pleural effusion | 0.96 (0.56–1.65) | 0.88 |
| Pneumothorax | 1.24 (0.36–4.27) | 0.74 |
| Subcutaneous emphysema | 0.30 (0.11–0.80) | 0.016 |
| IVC size (Ref: normal range [7–21 mm]) |  |  |
| Dilated (≥21 mm) | 1.23 (0.68–2.23) | 0.50 |
| Collapsed (≤7 mm) | 0.53 (0.28–1.02) | 0.06 |

Four patients who were transferred to a tertiary hospital for ECMO were excluded from the analysis (n=578).

Abbreviations: ARDS, acute respiratory distress syndrome; BMI, body mass index; CTR, cardiothoracic ratio; ECMO, extracorporeal membrane oxygenation; IVC, inferior vena cava; LA, left atrium; MV, mechanical ventilation; OR, odds ratio; PA, pulmonary artery; P/F ratio, partial pressure of oxygen/fraction of inspired oxygen ratio; RA, right atrium; SOFA, Sequential Organ Failure Assessment.
